# Increased Frequency of Hand Hygiene and Other Infection Prevention Practices Correlates with Reduced Surgical Wound Infection Rates in Spinal Surgery during the COVID-19 Pandemic

**DOI:** 10.3390/jcm11247528

**Published:** 2022-12-19

**Authors:** Andrea Perna, Francesco Maruccia, Franco Lucio Gorgoglione, Felice Barletta, Raffaele Vitiello, Luca Proietti, Francesco Ciro Tamburrelli, Domenico Alessandro Santagada

**Affiliations:** 1 Department of Orthopedics and Trauma Surgery, Fondazione Policlinico Universitario A. Gemelli IRCCS, 00168 Rome, Italy; 2 Department of Orthopedics and Trauma Surgery, Università Cattolica del Sacro Cuore, 00168 Rome, Italy; 3 Department of Orthopedics and Trauma Surgery, Fondazione Casa Sollievo Della Sofferenza IRCCS, San Giovanni Rotondo, 71013 Foggia, Italy

**Keywords:** hand hygiene, COVID-19, surgical wound infection, spinal surgery, hospital infections

## Abstract

Background: Due to the COVID-19 pandemic outbreak, many changes were done in the hospital practice, and new guidelines were issued in order to contain the infection spread. One of the most common measures is represented by a correct and frequent hand washing. Recently, an association between increased adherence to hand hygiene (HH) protocols and reduction in hospital infections was documented however no studies about the surgical wound infection rate were reported in the Literature. Methods: The present study represents a multicentric retrospective epidemiological study. The HH compliance rate was recorded through direct observations by trained nurses, 24 h a day. The primary outcome was HH compliance rate. The association of HH with spinal surgical wound infections was the secondary outcome. Results: We reported a compliance to HH practices during the pandemic period of 85.2% compared with 57% observed during 2019. Our analysis showed an overall surgical wound infection reduction of 66.6% during the hospital stay in the pandemic period. Conclusion: Hand hygiene has always been considered one of the most effective, reproducible and low-cost weapons to deal with hospital infections. The good health habits acquired during the COVID-19 pandemic should be maintained even after the virus is eradicated.

## 1. Introduction

One of the most common hospital acquired infections is represented by surgical site infection. Often they occur in the immediate postoperative period and could cause an increase in hospital stay, the need for antibiotic therapy and sometimes re-operations [1]. Despite the attention on sterility in the operating room and during surgical wound dressings, wound infection continues to be a cause of concern for spinal surgeons [1]. Hand hygiene (HH), consisting in hand washing with soap/chlorhexidine and water or hydroalcoholic solutions, following the five moments of hand hygiene, as recommended by WHO, represents a core practice in hospital infections prevention [2,3]. However, some studies in Literature showed poor adherence to this practice by the healthcare workers [4].

Since December 2019, a novel coronavirus has spread Worldwide starting from the city of Wuhan, Hubei Province, China. So far (March 18), according to WHO there are 121 millions total confirmed cases and 2.68 millions deaths due to SARS-CoV-2. In Italy, on 18 March 2020, there were 3.26 millions cases of COVID-19 disease and 103,432 deaths [5,6,7].

Due to the COVID-19 pandemic outbreak, many changes were done in the hospital practice, and new guidelines were issued in order to contain the infection spread and manage the emergency [8,9]. These include the ban on visits by relatives, the use of personal protective equipment, negative pressure isolation rooms and the reduction of elective surgical orthopedic activity in favor of conservative treatment [10,11]. One of the most common suggested measures is represented by a correct and frequent hand washing [2,3]. Despite a post-surgical complications rate during the COVID-19 pandemic was observed, particularly in elderlies [7,12,13], recently, an association between increased adherence to hand hygiene protocols and reduction in hospital infections was documented by Roshan et al. [14]. However no studies about the surgical wound infection rate were reported in the Literature. Therefore we decided to evaluate the HH compliance and its impact on infection rate of surgical wounds during the pandemic period and compare it with the same period in 2019.

## 2. Materials and Methods

### 2.1. Study Design

The present investigation represents a retrospective multi-center epidemiological analysis regarding all patients who underwent primary spinal surgery between 1 March and 1 October 2020 (pandemic period).

The study involves 2 orthopedics and traumatology departments respectively of a 2nd level trauma center (Fondazione Policlinico Universitario “A. Gemelli” IRCCS, Rome, Lazio, Italy) and a 1st level trauma center (Fondazione Casa Sollievo della Sofferenza IRCCS, San Giovanni Rotondo, Puglia, Italy) for a capacity of 100 beds and an estimated number of hospitalizations exceeding 3000 per year.

### 2.2. Variables

The primary outcome was HH compliance rate during pandemic period. The association between HH compliance and spinal surgical wound infections during the hospital stay was the secondary outcome. Surgical site infections were recorded and defined according to the standard of the centers for disease control and prevention (CDC) [15].

### 2.3. Institutional Database and Data Collection

Using the institutional databases for each patients enrolled were extracted: age, sex, comorbidities, American Society of Anesthesiologists (ASA) score, perioperative complication, hospital stay, need of blood transfusion, need of postoperative intensive care, surgical wound infection rate, location of surgery and microbiological isolation.

Finally, data collected was compared between COVID 19 pandemic and the same period of time (from 1 March to 1 October) in 2019 at our institutions (pre-pandemic period).

### 2.4. Participants and Eligibility Criteria

All patients who underwent surgical open instrumented spine procedure during the study period were potentially eligible for the study. Exclusion criteria were: (I) Metastatic and/or primary vertebral lesions; (II) Previous spinal surgery; (III) Previous spinal infections; (IV) Spinal infection before surgery; (V) Rheumatologic disease or immunosuppression condition (e.g., AIDS, organ transplantation), (VI) percutaneous procedures, (VII) Local use of antibiotics.

### 2.5. Pre and Post-Operative Routine

Thirty minutes before incision, a single preoperative dose (2 g) of cefazolin was used as antimicrobial prophylaxis. In all patients skin disinfection was performed using a solution of chlorhexidine gluconate 20 mg/mL and isopropyl alcohol 0.70 mL/mL (ChloraPrep). After wound closure with not-absorbable 2-0 (Polyethylene terephthalate, MERSILENE, Johnson & Johnson MedTech) stitches a sterile dressing was applied on the wound. Urinary catheter was removed on the 1st day after surgery; drainage was removed on the 2nd day after surgery. Mobilization and physiotherapy began the day after surgery. Surgical wound dressing was performed every 2 days during the hospital stay and then every 3 days after discharge. These procedures were followed in both (pre pandemic and pandemic) periods of the study.

### 2.6. HH Observation

The hand hygiene compliance rate among health workers of the two involved institutions was recorded through direct observations by nurses trained on the WHO direct observation method, 24 h a day. The direct observation technique was described in the WHO Hand Hygiene Technical Reference Manual (HHTRM) [15]. According to the HHTRM methodology, only healthcare workers in direct contact with patients are the target of observation. Observations data were collected anonymously. When the observation covers all the healthcare workers in a medical department usually a randomization method was preferred. The methodology adopted proposes sequencing the observation in sessions of limited duration, with each session being conducted in a different setting, with different healthcare workers and at different times. This will generally ensure a representative sample [15]. On average 25 observations per day were performed by trained observers. The nurses who performed the observations were tasked by the nursing coordinator and were involved in the normal clinical activities of the ward. All observations on HH compliance were covert. Data were registered and stored in a dedicated database according the institutional protocols. The data were then retrospectively analyzed.

The overall compliance was calculated by dividing the number of observed HH actions performed when an opportunity occurs, by the total number of opportunities. Generally, a HH compliance of  50% or higher was considered a sufficient compliance [16].

Monitoring HH compliance has been a long-established practice in our institutions. The data was collected and a report was published each month for employees of the department. In our retrospective study we analyzed these previously collected data on HH compliance and correlated with the rate of infections reported in the pre-pandemic and pandemic periods. The method used for the surveys is the same in the 2 periods, according to the institutional protocols.

### 2.7. Statistical Analysis

Data collected by the trained nurses were used to fill in the standard WHO observation proforma [13]. Chi square test was used to compare the differences in proportions. Spearman’s rank correlation coefficient was used to evaluate if HH compliance had a significant correlation with surgical wound infection rate. The significance was established for a value of *p* < 0.05. Dedicated software (GraphPad Software—Prism 8 for Mac) was employed. Only one decimal digit was reported, rounded up.

## 3. Results

### 3.1. HH Compliance

A total of 276 healthcare workers who worked in the units analyzed were evaluated for HH compliance. These were 85 nurses (30.6%), 57 doctors (20.5%), 43 medical students (15.5%), 59 resident doctors (21.2%), 40 nursing students (14.4%) and 32 health care assistants (11.5%) who worked in the units analyzed when the HH compliance assessment was carried out. A total of 3760 opportunities for HH were observed; overall compliance was 3203/3760, 85.2% (95% CI, 79–92) during the pandemic period while the overall compliance was 2610/4579, 57% (95% CI, 53–62) during pre-pandemic period. The HH compliance rate increased significantly during the pandemic period mostly due to HH compliance in World Health Organization moments 3 to 5 (r = 0.84, *p* = 0.002). The findings relative to HH compliance were resumed in Table 1.

### 3.2. Surgical Wound Infections

During the pandemic period 374 patients were surgically treated for spinal pathologies in the two centers involved in the study. Among these, only 289 met the inclusion criteria. During the pre-pandemic period 576 patients were surgically treated and 392 met the inclusion and exclusion criteria. A total of 3 cases of surgical wound infection (1%) was observed during pandemic period; during pre-pandemic period 9 cases of surgical wound infection (3.2%) was observed and this difference was statistically significant (RR = 2.23, OR = 2.19, *p* = 0.003). Both the groups of patients, pandemic and pre-pandemic, were comparable in terms of age, sex and ASA grade.

An overall surgical wound infection reduction of 66.6% between pre-pandemic and pandemic period was observed. Noteworthy, however, is that the surgical in the involved units during the pandemic with respect to 2019 (289 vs. 382). Furthermore using Spearman’s rank correlation coefficient, a strong inverse correlation between surgical wound infection rate and HH compliance by healthcare workers was found (−0.879, 95% CI = 0.865–0.897, *p* = 0.026). All surgical wound infections were treated with specific antibiotic therapy after pathogen isolation. Only 1 patient belonging to the pre-pandemic group needs surgical revision of the wound. Demographics features of patients were resumed in Table 2.

### 3.3. Location and Microbiology

During the pandemic period all 3 cases of surgical wound infection were observed in the lumbar spine. During the pre-pandemic period, among the 9 cases recorded, 6 (66.6%) were localized in the lumbar spine and 3 (33.4%) in the thoracic spine. About the ethology of infection we found 2 cases of Staphylococcus aureus and 1 case of Staphylococcus aureus Methicillin-resistant (MRSA) during the pandemic period. On the other hand, during the pre-pandemic period we found 5 cases of MRSA infections, 2 cases of Staphylococcus aureus Meticillim-sensitive (MSSA) infections and 2 cases of polymicrobial Gram-negative bacteria (Enterococcus, E. coli).

### 3.4. Subgroup Analysis

During the pandemic period in 87% of cases HH was performed by alcohol based hand rub, while in 13% of cases was performed by soap and water hand wash. During the pre-pandemic period indeed in only 34% of cases HH was performed by alcohol based hand rub. Stratifying by healthcare workers category, HH compliance ranged from a minimum of 31% among healthcare assistants and a maximum of 91% among medical students (Table 1).

## 4. Discussion

Although many studies have been done on the economic and social burdens that the current pandemic has placed on healthcare workers and patients [17], only a few researches have been done on the impact that increased hygiene practices and increased social distancing caused by the pandemic may have had on the incidence and control of surgical wound infections.

Early surgical wound infections during hospital stay represents one of the healtcare associated infections. The incidence of surgical wound infections varies in different structures and in different disciplines. It depends on multiple factors such: as the complexity and duration of the surgery, the site of surgery, the disinfection methods used in the operating room, preoperative antibiotic prophylaxis and local practices. The burden of surgical wound infections on healthcare and patients was studied in terms of length of hospital stay and functional outcomes. In fact, some authors argue that patients who have reported early surgical wound infection have more pain, a worse quality of life and poorer Oswestry function outcomes as well as a longer length of stay on average with respect to patients without wound infection [18]. Since surgical wound infections have multifactorial causes, it is difficult to design a study that analyzes all the factors involved together. Healthcare worker’s hands represent one of the main carriers of transmitting bacteria, viruses, and microorganisms. HH nowadays represent one of the fundamental means for the prevention of surgical wound infections [19]. Over the past few years, great efforts have been made to improve HH compliance around the world thanks to WHO.

Losurdo et al. [20] in a recent study on 541 surgical patients (among these 123 being from COVID-19 pandemic cohort), sustained that the rate of surgical wound infection was lower in patients of pandemic cohort with respect to pre-pandemic cohort. They found that this result could be correlated to the measures taken by the hospital to prevent the spread of COVID-19 infection (e.g., use of surgical masks, use of gloves, greater HH compliance, limitation of visits from relatives, etc.).

Other researchers [21] evaluated the impact of a hand hygiene new standard, emphasizing the use of alcohol gel for hand scrubbing before patients contact in a 600-bed plastic surgery ward. They found a substantial reduction of hospital surgical wound infection compared to previous years. Many authors support these findings and sustained that high compliance with HH could reduce the surgical wound infections rate in many surgical specialties [22,23,24].

On the other hand, some authors on a series of over 20,000 total joint arthroplasty found no difference in the rate of early and late infections between the pre-pandemic and pandemic study cohorts despite the greater compliance with HH during COVID-19 period [25].

In our investigation we found a significantly higher HH compliance in the pandemic period than in the pre-pandemic period (85.2% vs. 57%). Surely this was facilitated by the greater presence of hydroalcoholic gel dispensers, greater attention from health workers and more careful institutional guidelines on HH. Greater compliance with HH correlated with a marked reduction in the infection rate of surgical wounds (66.6%), despite the fact that surgical activity decreased during the pandemic period. It is noteworthy that healthcare assistants had the lowest HH compliance in both the pandemic and pre-pandemic periods. Similar results were observed by Novoa et al. [16]. In fact, they highlighted how the healthcare workers who had a lower compliance to HH were represented by the nurse assistants. Therefore, education and hand hygiene programs should continue to be undertaken for all classes of health workers to improve HH compliance.

### Limitations

The present study had some limitations. In fact, this represents a retrospective study and the sample size analyzed is relatively small. Therefore, further comparison studies with larger case series and a prospective design are necessary to strengthen our data. Furthermore, during the pandemic period, various infection prevention measures—Such as mask wearing, increased availability of alcoholic gel dispensers, use of gloves, limitations of visit by relatives could contributed to reduced infection levels, and this could represent a bias.

## 5. Conclusions

Hand hygiene has always been considered one of the most effective, reproducible and low-cost weapons to deal with hospital infections. And this has been confirmed, during the COVID-19 pandemic, by the observed hospital infection reduction trend simply after the adherence to hand hygiene protocols. The good health habits acquired during the COVID-19 pandemic should be maintained even after the virus is eradicated.

## Figures and Tables

**Table 1:** Hand hygiene (HH) compliance among the healthcare workers (HCWs).

	Pandemic Period	Pre-Pandemic Period	*p* Value
Number of HCWs	276	276	
HH Compliance			
Doctors (57)	68.1%	51.2%	0.002
Resident doctors (52)	77.4%	65.3%	0.003
Medicine Students (43)	91%	71.1%	0.001
Nurses (85)	89.5%	68.5%	0.002
Nurses students (40)	87.7%	65.2%	0.003
Health care assistants (32)	31%	27%	0.876
Total	85.2%	57%	0.002

**Table 2:** Demographics features of patients underwent to instrumented spinal surgery.

	Pandemic Period	Pre-Pandemic Period	*p* Value
Number of patients	289	392	
Gender	157 F, 132 M	221 F, 171 M	
Age	64.3 (+/−7.8)	65.2 (+/−9.1)	0.768
BMI	26.6 (+/−2.2)	27.2 (+/−1.8)	0.643
ASA Score			
- I	56 (19.6%)	77 (19.6%)	0.739
- II	159 (55.6.%)	194 (49%)	0.613
- III	74 (24.8%)	121 (30.2%)	0.578
Comorbidity			
AF in ONA	13 (4.6%)	21 (5.3%)	0.836
Hypertension	79 (27.7%)	101 (25.3%)	0.782
Obesity	15 (5.3%)	23 (5.8%)	0.851
COPD	9 (3.2%)	17(4.3%)	0.793
Hypothyroidism	18 (6.3%)	38 (9.5%)	0.781
Diabetes	16 (5.5%)	25 (6.3%)	0.698
Smokers	97 (34%)	141 (35.3%)	0.817
Surgery level			
Cervical	37 (12.9%)	48 (12%)	0.834
Thoracic	76 (26.6%)	124 (31%)	0.759
Lumbar	176 (61.6%)	220 (55%)	0.693
Surgical approach			
Anterior	39 (13.3%)	57 (14.5%)	0.865
Posterior	198 (69.3%)	240 (60%)	0.841
Combined	52 (18.2%)	95 (23.7%)	0.872
Wound infection	3 (1%)	9 (3.2%)	0.003

AF: Atrial Fibrillation; ASA: American Society of Anaesthesiologists; BMI: Body Mass Index; COPD: Chronic Obstructive Pulmonary Disease; ONA: Oral New Anticoagulant (Dabigatran, Rivaroxaban Apixaban).

## Data Availability

Data relating to this article are available from the corresponding author upon reasonable request.
